# The androgen receptor plays a suppressive role in epithelial- mesenchymal transition of human prostate cancer stem progenitor cells

**DOI:** 10.1186/s12858-015-0042-9

**Published:** 2015-05-06

**Authors:** Ma Zhifang, Wei Liang, Zhang Wei, Hao Bin, Tu Rui, Wu Nan, Zhang Shuhai

**Affiliations:** Department of Urology, First Hospital of Shanxi Medical University, Taiyuan, 030001 China

**Keywords:** Prostatic neoplasms, Stem progenitor cell, Epithelial-mesenchymal transition, Androgen receptor

## Abstract

**Background:**

To investigate the roles of androgen receptor (AR) in epithelial- mesenchymal transition (EMT) in human prostate cancer stem progenitor (S/P) cells isolated from LNCaP cell line.

**Methods:**

The S/P cells were obtained from LNCaP cell line through florescence-activated cell sorting (FACS). AR was overexpressed in S/P cells through lentivirus. Western blot assay was used to detect the EMT markers expression, such as E Cadherin, N Cadherin, Vimentin and Snail. MTT assay, soft agar colony formation assay, sphere formation assay and migration assay were used to investigate AR’s roles in EMT of S/P cells. Cell signaling pathways associated with proliferation and apoptosis of S/P cells were detected simultaneously. And S/P cells were treated with in vitro combinatory use of LY 294002 (inhibitor of AKT signaling molecules) with γ-TT and/or 5-AZA.

**Results:**

Our data showed that S/P cells from LNCaP had high EMT markers expression, more tumorigenesis and strong migration ability. And in S/P cells overexpressed with AR, the expression of EMT markers decreased. In addition, these cells had less proliferation ability, tumorigenesis ability, self-renewal and migration ability. At the same time, targeting S/P cells with AKT signaling pathway inhibitor LY29004 andγ-TT and/or 5-AZA could inhibit S/P cell’s proliferation and tumorigenesis.

**Conclusions:**

Our data suggest that AR played a negative role in EMT of PCa S/P cells, by regulating AKT cell signaling pathway, which could be a new strategy to treat castration resistant prostate cancer (CRPC).

## Background

Prostate cancer is the most common malignancy in the world and the second most common cause of cancer-related mortality in men [1]. Early prostate cancer (T1-T2) can undergo radical surgery or radiation therapy, the curative effect is good. For locally advanced or metastatic prostate cancer (T3-T4), endocrine therapy is the preferred method. Unfortunately, after 1–3 years, the tumors ultimately progress and become castration resistant prostate cancer (CRPC). This is the end stage of prostate cancer and is the bottleneck of treatment. The mechanism of CRPC advance, why the tumor is not sensitive to chemotherapy, was not completely clear.

More and more evidence indicate that the cancer stem cells (CSC) exist objectively and play an important role in the tumorigenesis and progression of the tumors [2,3]. This part takes up only a small percentage of all cancer cells, but is closely related to tumor recurrence and metastasis. Many research has shown that cancer drug resistance to chemotherapy is associated with CSC, which have the potential for self-renewal, differentiation, strong migration and invasion ability [4, 5]. Cell signaling pathways related to maintain stem cell self-renewal and proliferation include PI3K/AKT, Wnt, STAT3/5, EGF/EGFR and so on [6-9]. Preliminary works from our research group showed that after endocrine therapy, the prostate cancer stem/progenitor (S/P) cells increased in tumor tissue of the patients, which further confirmed the role of S/P cells in prostate cancer progression [10].

The epithelial- mesenchymal transition (EMT) is the process that in a particular physiological and pathological conditions, the epithelial cells transfer to mesenchymal cells, involving in multiple genes and multi-step, the intercellular adhesion weakening and cell movement strengthening. EMT provides such a basis for epithelial tumor cells. Lue’s research [11] had shown that a zinc transporter LIV1 could promote EMT and metastasis of prostate cancer cells. This procedure is mediated through ERK signaling pathway. Other studies have found that BMP7 and SIRT1 could induce EMT in prostate cancer PC-3 cells, and PI3K and ERK signaling pathway was involved in this process. This promoted invasion and metastasis of prostate cancer [12,13]. In addition, the EMT markers can be detected in prostate cancer patients, with primary tumors and bone metastases. Immunohistochemical study showed that the expression of EMT markers was higher in the edge location cells of primary tumors and metastatic lesion than that of the cells in the center of the tumor. Notch1 expression in bone metastases is significantly higher than that in primary tumorsand, and may play an important role in the bone metastasis of prostate cancer [14]. These data suggest that EMT plays an important role in the invasion and metastasis of prostate cancer. Consistent with this, our preliminary data showed the cancer cells with EMT phenotype increased after endocrine therapy in human PCa tissue [15,16].

It was shown that EMT phenotype tumor cells had certain features of stem cells, and some stem-like cells had EMT features, and these two types of cells were associated with tumor drug resistance [17-19]. Androgen receptor (AR), a member of the nuclear receptor super family, can be activated by its ligands, androgens, to regulate its target gene expression. Androgen/androgen receptor (AR) signaling plays pivotal roles in the prostate development and homeostasis as well as in the progression of prostate cancer (PCa) [20]. Whether prostate cancer stem cells have the features of EMT and roles of AR in this process was unclear, in this study, we would investigate EMT characteristics in prostate cancer S/P cells, and the roles of AR in regulating EMT and characteristics of S/P cells.

## Methods

### Cell lines, transfection and infection

The human PCa cell lines LNCaP were purchased from the Chinese Type Culture Collection (CTCC). LNCaP (passage X) was maintained in a humidified incubator (95% O_2_; 5% CO_2_) and cultured with RPMI 1640 medium (Invitrogen, Carlsbad, CA) with 10% fetal calf serum and 2 mM glutamine (Gibco BRL), 1% penicillin and streptomycin (Invitrogen). Androgen deprivation or androgen treatment in vitro were carried out by culturing the cells in medium containing 10% charcoal-stripped fetal calf serum plus 1 nM 5α-dihydrotestosterone (DHT, Sigma) or 10 nM DHT. For immunocytochemical staining, cell lines were grown on chamber slides (Thermo Fisher Scientific Inc. MA) until confluence.

For transfection of plasmid pRetrosuperMycshRNA (Addgene, MA) and Oligo Bcl-2 siRNA (Santa Cruz Biotechnology, CA), cells were plated in culture dishes 24 hours before transfection and 2 days later in accordance with the manufacturer’s instructions using Lipofectamine Plus reagent (Invitrogen Life Technologies, CA). After transfection, cells were used to do MTT assay. The scrambled siRNA (Santa Cruz Biotechnology, CA)) was used as the negative control (sc-37007).

For infection of lentivirus carrying vector or AR, 293 T cells were transfected with a mixture of DNAs (Lentiviral vectors pWPI) (Addgene, MA) containing AR (psPAX2 packaging plasmid, and pMD2G envelope plasmid, at a 4:3:2 ratio) using Lipofectamine (Invitrogen, CA) according to the manufacturer’s protocol. After infection, the media containing the virus was changed into normal culture medium. Since the pWPI vector contains GFP, transfection efficiency could bel monitored by detecting GFP fluorescence.

The research had been performed with the approval of the ethics committee of First Hospital of Shanxi Medical University; written informed consent for participation in the study was obtained from participants.

### Antibodies

The antibodies c-Myc, MAPK (Erk1/2), Phospho - MAPK (Erk1/2), Stat3, Phospho - Stat 3, AKT and Phospho - AKT were obtained from cell signaling technology, MA, USA. The antibodies P63, AR, Integrin β1, Bcl-2, GAPDH, Wnt-1(G-19) and TGFβ1 were derived from Santa Cruz Biotechnology, CA, USA. The antibodies CD133, CK8, E Cadherin, N Cadherin, and Snail were derived from Abcom, MA, USA. The antibody CK5 was obtained from Covance, CA, USA. The antibody CD44 was derived from eBioscience, CA, USA. The antibody Vimentin was derived from Fisher Scientific, MA, USA. The antibody Ki67 was obtained from Novocastra, Newcastle, United Kingdom.

### Florescence-activated cell sorting (FACS)

For flow cytometry, cells were dissociated with Accutase (Innovative Cell Technologies, CA.) and washed twice in the staining solution containing Ca^2+^- and Mg^2+^-free PBS with 1 mM EDTA, 25 mM Hepes (pH 7.0) (Gibco BRL), and 1% FBS. Cells were stained live in the staining solution containing conjugated anti-CD44, and anti-CD133 antibody (50 min at 4°C). Samples were analyzed on a BD LSR II flow cytometer (Becton Dickinson Immunocytometry Systems, CA). A minimum of 500,000 viable cell events were collected per sample. For sorting, 2 × 10^7^ cells were processed for CD44 and CD133 multi color staining, along with appropriate negative controls and single-color positive controls. The CD44+/CD133+ population was sorted out on a BD FACS Diva cell sorter (Becton Dickinson Immunocytometry Systems, CA, USA).

### RNA extraction and quantitative real-time PCR analysis

RNA was extracted using the Trizol reagent (Invitrogen), and first-strand cDNA was synthesized from 1 μg of total RNA in 20 μl reactions containing RT buffer with dNTPs, Oligo-dT, RNase inhibitor, and Moloney murine leukemia virus reverse transcriptase according to the manufacturer’s protocol (Invitrogen). Reverse transcription reactions (used BIO-RAD MyCycler thermal cycler) were incubated at 25°C for 5 min and then 42°C for 30 min, followed by 85°C for 5 min, and aliquots of the reaction products were used in later quantitative real-time PCR analysis. The quantitative PCR analysis was done using BIO-RAD C1000 Thermal cycler system and the SYBR green PCR master mix kit (Applied Biosystems), according to the manufacturer’s suggestions. The housekeeping gene, β-actin was used for normalization. Relative mRNA expression was calculated as described. The following primer pairs were used: Integrin α2β1 forward, 5-GTCGGGGGCTTCAACTTAGAC-3; reverse, 5-CCTGGCTGGCTGGTATTAGC-3; CD133 forward, 5-AGCCTTCATCCACAGATGCT-3; reverse, 5-GTGCATTTCTCCACATTT-3; CK 5 forward, 5-GTGATGCTGAAGAAGGATGTAG-3; reverse, 5-TCC AGG TTG CGG TTG TTG-3; CK 8 forward, 5-TGG AGT CTC GCC TGG AAG-3; reverse, 5-CCT CGT ACT GTG CCT TGA C-3; CD 44 forward, 5-TTT GCA TTG CAG TCA ACA GTC-3; reverse, 5-GTT ACA CCC CAA TCT TCA TGT CCA C-3; PSA forward, 5-GGT GAC CAA GTT CATGCT GTG-3; reverse, 5-GTG TCC TTG ATC CAC TTC CG-3; AR forward, 5-TAG CCC CCT ACG GCT ACA; reverse, 5-TTC CGA AGA CGA CAA GAT GGA C-3; β-actin forward, 5-CAT GTA CGT TGC TAT CCA GGC-3; reverse, 5-CTC CTT AAT GTC ACG CAG AT-3; All samples were run in triplicate.

### Western blots

Detailed procedures had been described previously [21]. Briefly, cells were exposed to RIPA lysis buffer (with protease inhibitor), and were incubated for 30 min on ice. The homogenate was centrifuged at 14,000 rpm for 10 min at 4°C. The protein supernatant concentration was determined using the Bradford assay from BIO-RAD Laboratories. Then the protein was prepared for electrophoresis run on SDS/PAGE gel and then transferred onto PVDF membrane (Pall Corporation, FL). After the membrane was blocked by 5% milk PBST, the first antibodies (against c-Myc, MAPK (Erk1/2), Phospho-MAPK (Erk1/2), Stat3, Phospho-Stat 3, AKT and Phospho – AKT, AR, Bcl-2, GAPDH, Wnt-1(G-19) and TGFβ1) were diluted in blocking buffer to appropriate concentrations and incubated with protein blots (on PVDF membrane) individually. Following reaction with HRP-conjugated secondary antibodies, the signals were developed with enhanced chemiluminescence (ECL) substrate (Thermo Scientific, IL). The band densities were digitized using ChemiDocTM XRS+ Imaging System (BIO-RAD Laboratories, CA); The GAPDH protein was used as internal control.

### Soft agar colony formation assay

To compare the different cells’ tumorigenetic ability, a cell suspension (1 × 10^4^ cells/well) in 2 mL of 0.5% low-melting SeaPlaque CTG agarose (Cambrex Bio Science Rockland) with RPMI 1640 was overlaid into 6-well plates containing a 1% agar base. The 2 mL normal medium was added on the upper cells contained agar. All samples were plated in triplicate. The plates were incubated at 37°C in a humidified incubator for 21 days. The colonies were visualized by staining with 1% crystal violet. The experiment was analyzed in triplicate, and colonies larger than 1 mm in diameter were counted.

### Migration and invasion assay

The migrative and invasive potential of different cells was assessed using the 8 μm polycarbonate membrane plate Transwell system (Corning Incorporated, NY). Cells (1 × 10^5^) were resuspended in 100 μl serum free medium and added in the upper Transwell compartment. 600 μl of 20% CS-FBS medium was placed in the lower compartment to act as an attractant. 1 or 10 nM DHT was added in the medium. After 24 hours incubation at 37°C and 5% CO_2_, the filters were fixed in methanol for 10 min at 4°C, stained with 1% toluidine blue (BIO-RAD, CA) for 5 min at room temperature and then washed carefully in ddH_2_O. Cells that had not migrated, were removed from the upper face of the membranes with cotton swabs. After air dried, the membranes were observed under light microscope. The average number of cells per field of view (five random fields per membrane) was counted at × 20 magnification. For invasion assay, before the cells were planted into the upper Transwell, 50 μl Matrigel was added on the membrane. The incubation time was prolonged to 48 hours. Each experiment was repeated at least three times and each time the mean numbers of migrating or invading cells were taken from three chambers.

### Sphere formation assay

Single cells suspension (1 × 10^3^, in 50 μl medium) was mixed with Matrigel 50 μl (keep on ice before use) and 100 μl of mixture was placed along the rim of the 24 well low attached culture plates (BD, CA). Every kind of cells was repeated three wells. The culture plate was incubated in 37°C incubator for 10 min and then 500 μl medium was added. Cells were grown in serum-free epithelial basal medium with 1 or 10 nM DHT, supplemented with Prosta Life Life Factors (Lifeline cell technology, Germany). Spheres were counted after 7 to 14 days under Olympus light microscope.

### Immunostaining

For culture cells immunofluorescence staining, the cells were fixed with ice cold methanol on ice for 15 min and then washed with PBS thrice. The cells were permeabilized with 0.1% Triton X-100/PBS at room temperature for 10 min and then washed with PBS. Non-specific antibody binding sites were blocked for 30 min with 5% bovine serum albumin PBS and not be washed. Cells were incubated with first antibodies diluted in blocking buffer at 20°C for 2 hours. Following 3 times PBS washes, second antibodies diluted in blocking buffer were added on the cells at room temperature for 60 min. After air-dried, the cells were covered by mounting solution (containing DAPI) and visualized with an Olympus IX70 fluorescent microscope. The separate images were obtained for Texas Red and DAPI fluorescence, and images were overlaid with Adobe Photoshop (Adobe Systems, CA). When the cells were double stained, the first antibodies should be from different resource.

### Cells proliferation assay

All kinds of cells were incubated in 12–96 well dishes (10^3^-10^5^ cells/well) for 6 days, 3-(4,5-dimethylthiazol-2-yl)-2,5-diphenyltetrazolium bromide (MTT; 0.5 mg/mL; Sigma) was added at days 0, 2, 4, and 6. After 2 hours of reaction, absorbance of the converted dye was measured at a wavelength of 570 nm with background subtraction at 650 nm.

### Statistical analysis

Data are shown as the mean ± SD. And statistical analysis was obtained using SPSS version 15.0computer software (SPSS Inc., Chicago, IL, USA). A P-value of <0.05 was taken to indicate statistical significance.

## Results

### Isolating S/P cells from LNCaP cell line

**Figure 1:**
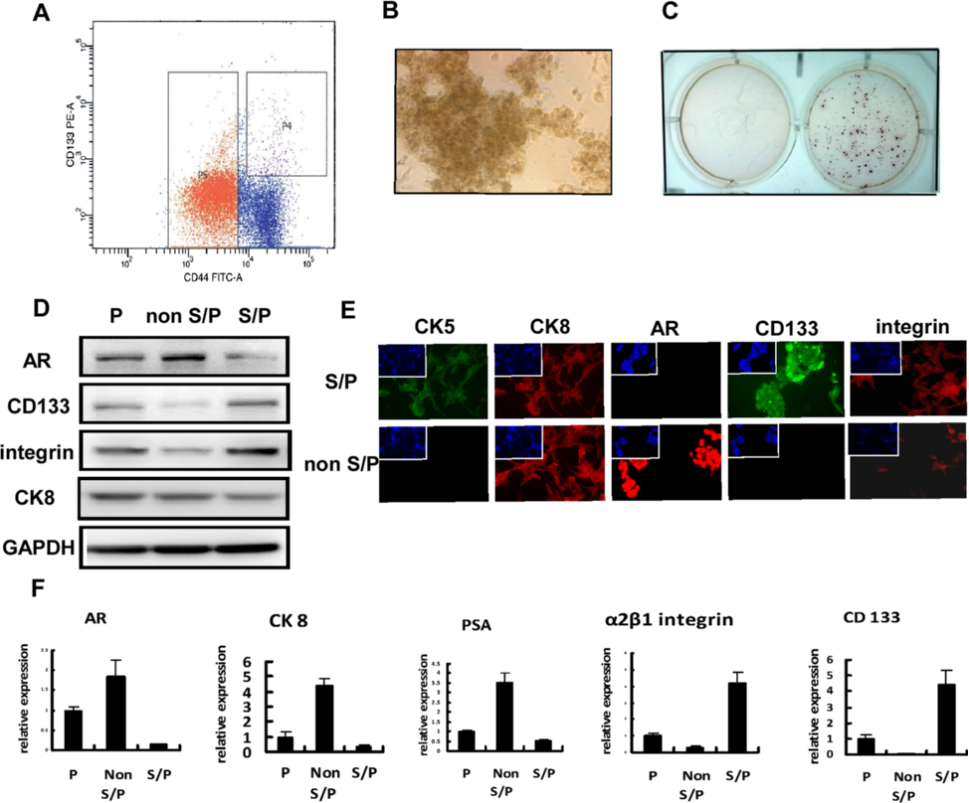
Isolating S/P cells from LNCaP cell line. **(A)** Flow cytometry isolation of S/P (1.5%) and non S/P (98.5%) cells. Cells were grown in regular RPMI medium containing 5% of FBS (cells of passage number less than 3 were used for all experiments). **(B)** Morphology of sorted S/P cells. **(C)** Soft agar assay of S/P and non S/P cells. **(D)** Western blot analysis of AR, CD133, Integrin β1, and CK 8 using cell extracts of parental, S/P, and non S/P cells. **(E)** IF staining results using antibodies of CK5, CK8, AR, CD133 and Integrin. **(F)** qRT-PCR analysis results of markers of parental, S/P, and non S/P cells.

The published paper by others and results of our laboratory’s recent studies indicated low expression level of AR in PCa S/P cells [22,23]. In order to investigate AR’s roles in EMT of PCa S/P cells, S/P cells were isolated from a single androgen dependent PCa cell lineLNCaP, using antibodies to CD133 and CD44. About 1.5% S/P cells were isolated using FACS (Figure 1A). And the morphology of sorted LNCaP S/P cells was shown in Figure1B. As shown in Figure 1C, the S/P cells had higher tumorigenicity than that of non S/P cells, when tested using soft agar colony formation assay. Then the stem cell markers expression of S/P cells was detected through Western blot analysis, immunofluorescence (IF) staining and qRT-PCR (Figure 1D-F). The S/P cells markers, such as CD133, integrin and CK5 were expressed higher than those of non S/P cells. Meanwhile, the mature epithelial cell markers in S/P cells, such as AR, PSA and CK8 were expressed lower than those of non S/P cells. These results suggested that the isolated small number side population was S/P cells with higher tumorigenicity.

### Identification of EMT characteristics in S/P cells from LNCaP cell line

**Figure 2:**
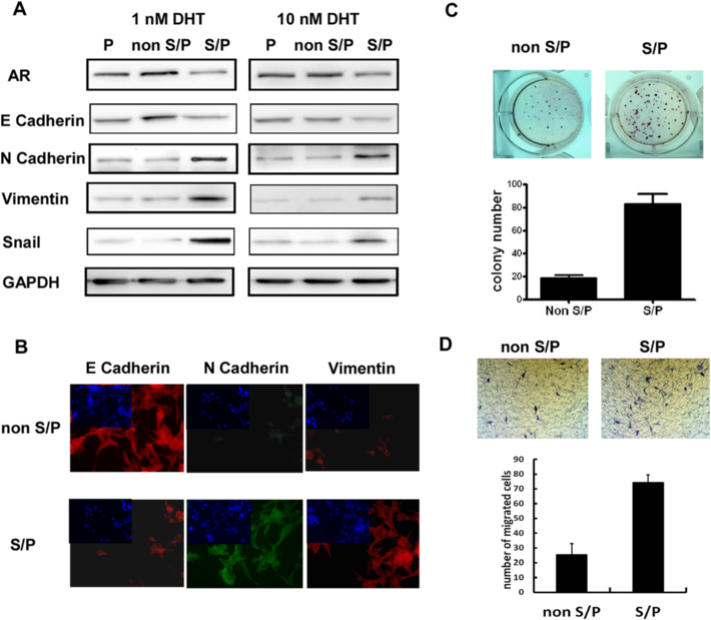
Identification of EMT characteristics in S/P cells from LNCaP cell line. **(A)** Western blot analysis of AR, E Cadherin, N Cadherin, Vimentin and Snail using cell extracts of parental, S/P and non S/P cells. **(B)** IF staining results using antibodies of E Cadherin, N Cadherin and Vimentin. **(C)** Soft agar colony formation assay. The plates were incubated at 37°C in a humidified incubator for 21 days. Colonies larger than 1 mm in diameter were counted. **(D)** Migration assay. The migrative potential of different cells was assessed using the 8 μm pore Transwell system. The average number of cells per field of view (five random fields per membrane) was counted at × 20 magnification.

Western blot analysis and IF staining were used to test EMT markers expressed in S/P cells from LNCaP cell line. Using cell extracts of parental, S/P and non S/P cellsAR, the EMT markers including E Cadherin, N Cadherin, Vimentin and Snailproteins were detected using Western blot analysis. N Cadherin, Vimentin and Snailin S/P cells were expressed higher than those in non S/P cells. However, epithelial cell marker E Cadherin was expressed low in S/P cells (Figure 2A). IF staining results demonstrated the similar thing (Figure 2B). As shown in Figure 2C and D, then soft agar colony formation assay and migration assay showed that S/P cells had higher tumorigenicity and migration ability, than those of non S/P cells. Our data suggested that S/P cells from LNCaP cell line showed EMT characteristics.

### AR was a suppressor in EMT of S/P cells from LNCaP cell line

**Figure 3:**
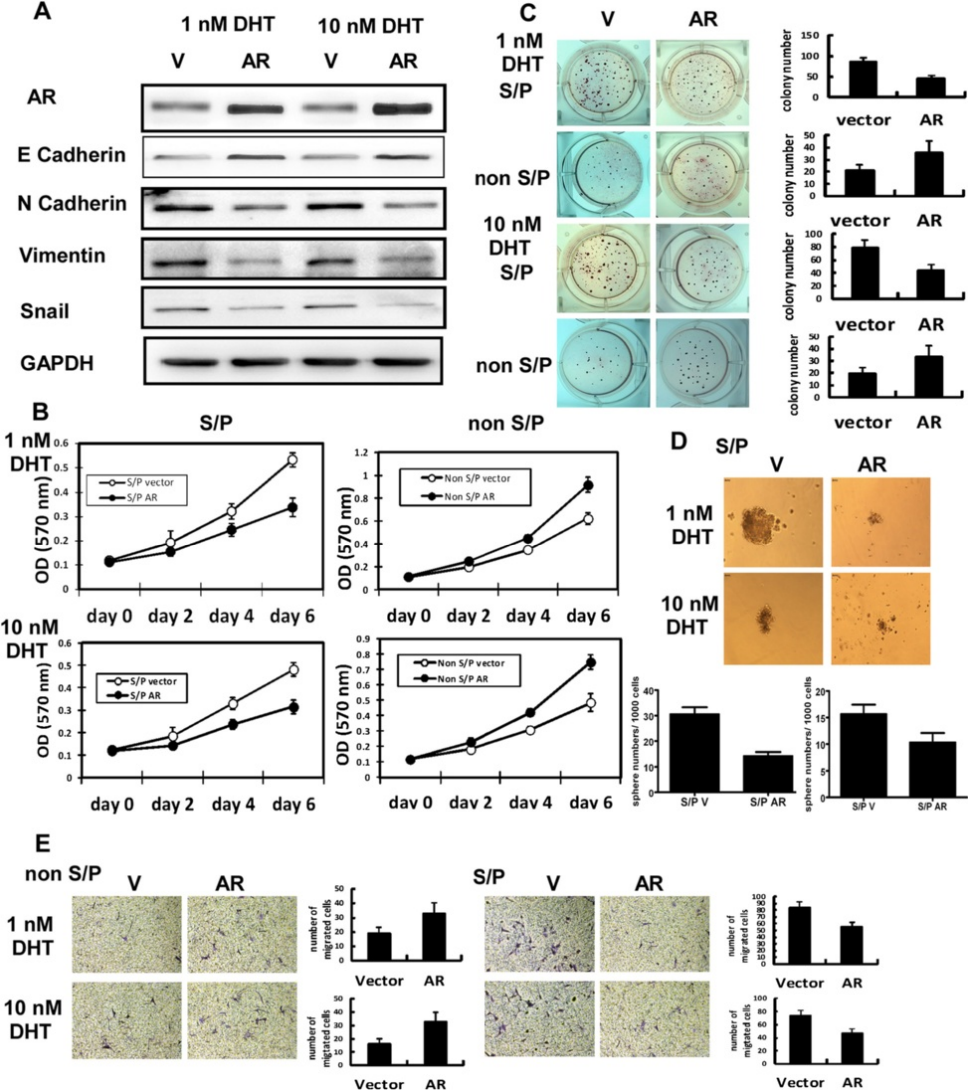
AR is a suppressor in EMT of S/P cells from LNCaP cell line. S/P Cells were infected with lentivirus carrying either vector or AR and grown in two different androgen concentrations, charcoal stripped (CS)-FBS + 1 nM DHT, CS-FBS + 10 nM DHT. **(A)** Western blot analysis of AR, E Cadherin, N Cadherin, Vimentin and Snail using cell extracts of vector S/P and AR S/P cells. **(B)** MTT assay. Cells were incubated in 24 well dishes (1 × 10^4^ cells/well). At 0, 2, 4, and 6 days, MTT assays were performed. **(C)** Soft agar colony formation assay. The plates were incubated at 37°C in a humidified incubator for 21 days. Colonies larger than 1 mm in diameter were counted. **(D)** Sphere formation assay. Cells (5,000) were mixed with Matrigel (1:1 ratio, total 200 μl) and placed in rim of well of 24well culture plates. After solidified, 1 ml of medium was added and grown for 21 days. Pictures were taken under microscope. **(E)** Migration assay. The migrative potential of different cells was assessed using the 8 μm pore Transwell system. Cells (1 × 10^5^) were resuspended in 100 μl serum free medium and added in the upper compartment. 600 μl of 20% CS-FBS medium was placed in the lower compartment. After 24 hours, cells that had not migrated were removed from the upper face of the membranes with cotton swabs. The average number of cells per field of view (five random fields per membrane) was counted at × 20 magnification. All experiments samples were plated in triplicate.

S/P Cells were infected with lentivirus carrying either vector or AR and grown in two different androgen concentrations, charcoal stripped (CS)-FBS + 1 nM DHT, and CS-FBS + 10 nM DHT. Western blot assay was used to test AR, E Cadherin, N Cadherin, Vimentin and Snail expression using cell extracts of vector S/P and AR S/P cells. As shown in Figure 3A, the expression of EMT markers decreased, when AR was overexpressed in S/P cells. Then how AR affected S/P cells proliferation, was investigated. Cells were incubated in 24 well dishes (1 × 10^4^ cells/well). At 0, 2, 4, and 6 days, MTT assays were performed. The ARS/P cells proliferation was inhibited during different DHT concentration, compared to that of Vector S/P cells. But AR played an opposite role in non S/P cells (Figure 3B). Soft agar colony formation assay was used to test tumorigenicity. After incubated for 21 days, colonies larger than 1 mm in diameter were counted. The ARS/P cells tumorigenicity was inhibited under different DHT concentration, compared to that of Vector S/P cells (Figure 3C). Similarly, the self-renewal ability was inhibited, when AR was overexpressed in S/P cells (Figure 3D). Moreover, the migrative potential of ARS/P cells was inhibited in different DHT concentration, compared to that of Vector S/P cells. Meanwhile, AR played an opposite role in non S/P cells (Figure 3E).

### The signaling pathways activation related to self-renewal/proliferation in LNCaP S/P cells

**Figure 4:**
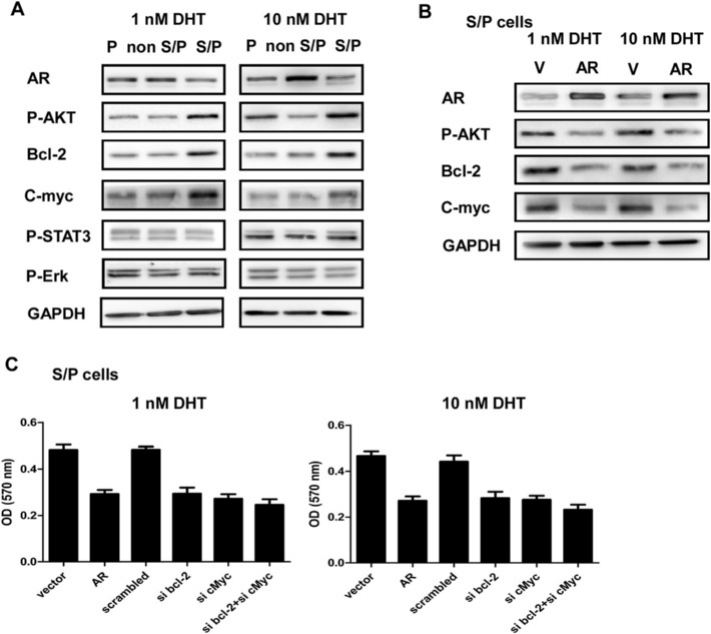
Activation of self-renewal/proliferation related signaling pathways in LNCaP S/P cells. **(A)** The probably related signaling pathways in parental, S/P, and non S/P cells of LNCaP cell line were examined by Western blot analysis, such as AKT, Bcl-2, c-Myc, MAPK (Erk1/2) and Stat3. **(B)** Cells (LNCaP S/P) were infected with lentivirus carrying V/AR and the levels of AR, p-Akt, bcl-2, and c-Myc were examined by Western blot analysis. **(C)** The S/P cells of LNCaP cell line were transfected with AR, Oligo bcl-2 siRNA, or/and c-Myc siRNA plasmids indicated. At the end of 4 days of culture after transfection, cells were used to do MTT assay.

It was known that PI3K/AKT, Bcl-2, c-Myc, MAPK (Erk1/2), TGF-ß, and Stat3 cell signaling pathways might involve in S/P cells proliferation and apoptosis. The probably related signaling pathways in parental, S/P, and non S/P cells of LNCaP cell line were examined using Western blot analysis. It was found that P-AKT, Bcl-2 and c-Myc were expressed higher in S/P cells, compared to those of non S/P cells (Figure 4A). When LNCaP S/P cells were infected with lentivirus carrying AR, the expression of P-Akt, bcl-2, and c-Myc were inhibited (Figure 4B). Then to confirm the signaling pathways related to S/P cells proliferation, the S/P cells of LNCaP cell line were transfected with AR, Oligo bcl-2 siRNA, or/and c-Myc siRNA plasmids indicated. At the end of 4 days of culture after transfection, cells were used to do MTT assay. It was found AR, sibcl-2, or/and sic-Myc all could inhibit S/P cells proliferation. Combining use of si bcl-2 with si c-Myc could show stronger inhibition (Figure 4C).

### In vitro combinatory use of LY 294002 with γ-tocotrienol (γ-TT) and/or 5-aza-2′-deoxycytidine (5-AZA) showed synergistic effect of killing PCa S/P cells

**Figure 5:**
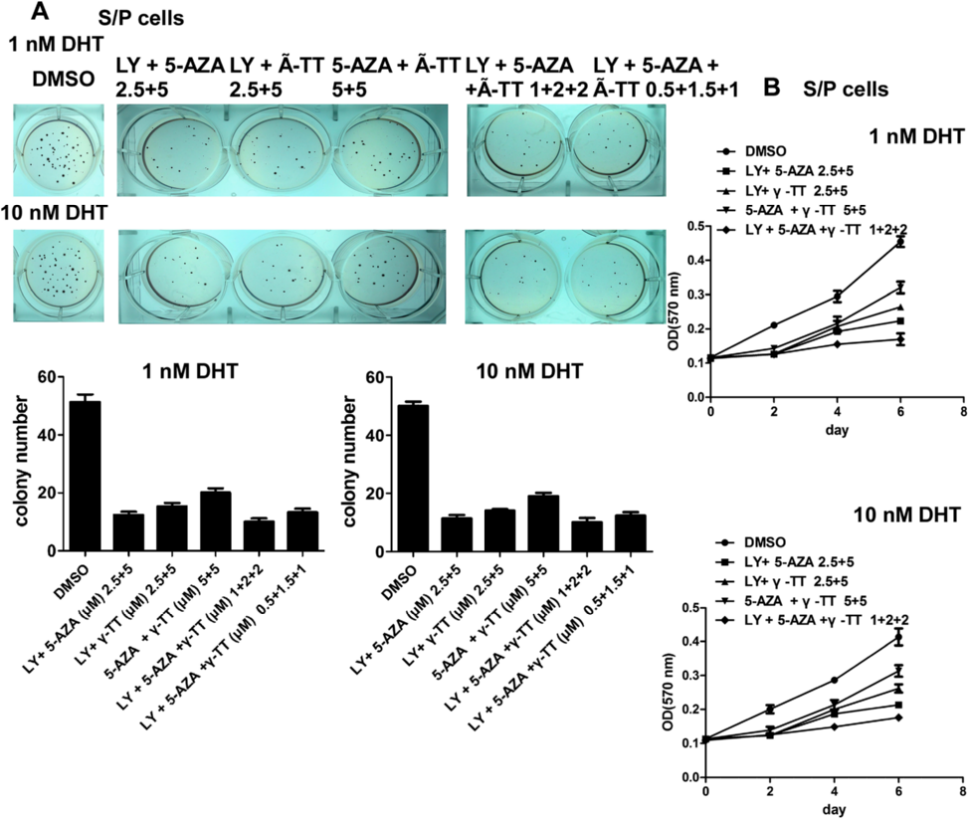
In vitro combinatory use of LY 294002 (inhibitor of AKT signaling molecules) with γ-TT and/or 5-AZA showed synergistic effect of killing PCa S/P cells. **(A)** Soft agar colony formation assay. A cell suspension (1 × 10^4^ cells/well) in 2 ml of 0.5% low-melting agar with RPMI 1640 was overlaid into 6well plates containing 1% agar base. The 2 ml normal medium was added on the upper cells contained agar. The plates were incubated at 37°C in a humidified incubator for 21 days. The colonies were visualized by staining with 1% crystal violet. Colonies larger than 1 mm in diameter were counted. **(B)** MTT assay. Cells were incubated in 24 well dishes (1 × 10^4^ cells/well). At 0, 2, 4, and 6 days, MTT assays were performed.

LY 294002 is an inhibitor of AKT signaling. γ-TT can suppress S/P cells through the interruption of targeting molecules including bcl-2 and Akt. 5-AZA is a de-methylating agent and can induce AR expression of S/P cells. LY 294002, γ-TT and 5-AZA were used in various combinations. Two or three reagents could suppress S/P cells tumorigenesis and growth. Combining use of three reagents could show stronger inhibition in soft agar colony formation assay (Figure 5A). At last, MTT assay was used to show that these reagents could suppress S/P cells growth. It was found that the combinational use of LY 294002, γ-TT and 5-AZA did leaded to a significant suppression of S/P cells proliferation (Figure 5B).

## Discussion

It’s believed that cancer stem/progenitor cells and EMT played very important roles in prostate cancer development [24]. EMT is closely related to PCa drug resistance. EMT and stem-like cell markers expression of the docetaxel-resistant PCa cells had increased. ZEB1 siRNA transfection reverted docetaxel resistance and reduced CD44 expression in DU-145R and PC-3R [25]. It was shown that PCaradio resistance is associated with EMT and enhanced CSC phenotypes via activation of the PI3K/Akt/mTOR signaling pathway [26]. After androgen-deprivation therapy (ADT), the S/P cells increases, which have low AR expression. It was reported that ADT could lead to EMT in both normal prostate and prostate cancer tissues [15]. Sethi [14] found that the cancer cells EMT marker expression was different at the edge of human primary tumors and bone metastasis of prostate cancer. The expression of EMT marker Vimentin, nuclear factor-кB (NF-кB), Notch-1 and ZEB1 increased. Meanwhile, epithelial markers E Cadherin expression level was low. This study suggested EMT was closely related to invasion and metastasis of prostate cancer. Another study showed that EMT phenotype could be detected in ARCaPM and PDGF-D overexpression PC-3prostate cancer cell lines. Compared with the ARCaPE and PC-3 cells, the tumor formation and self-renewal capacity of these cells significantly increased. Stem cell markers Sox2, Nanog, Oct4, Lin28B and/or Notch1 expression significantly increased and the cells had some characteristics of stem cells. MiR200 could inhibit Notch1 expression, and cells tumor formation capacity and self-renewing ability decreased. Through Notch1 signaling pathway, MiR200 regulated characteristics of stem cells of EMT phenotype prostate cancer cell [27]. All these results indicated that EMT has some relationship with CSC.

Our preliminary data show that both tumor cells with EMT markers and tumor cells with CSC markers increase after ADT in tumor tissue of prostate cancer patients [10, 16]. Stem-like SP cells acquired more complete EMT molecular features and exhibited stronger aggressive capability [28]. In this research, our group made it clear that CSC of prostate cancer had some EMT characteristics. Compared with those of non S/P cells, the EMT markers expression of S/P cells increased, tumorigenesis ability and migration and invasion capacity wre enhanced in S/P cells. These two kinds of cells increased, after ADT and these may common led to the failure of traditional endocrine treatment and disease progress.

Androgen/androgen receptor (A/AR) axis cancer [29]. Now it was considered that prostate cancer cells varied, and are mainly luminal cells, but there are also some basal cells and intermediate cells. Some basal cells with CSC markers may be the origin of the tumor. Recent studies have shown that AR expression level was different in prostate cancer cells, played a different role [30-32]. The current research and our work showed that AR expression was low in prostate cancer S/P cells. Transferring ARintoS/P cells could inhibit their proliferation. AR’s roles in EMT characteristics of S/P cells and related regulatory mechanism were not clear. In this study we transfered AR into S/P cells with lentivirus. Compared with that of vector S/P cells, using different methods of immune detection, it was found that the expression of EMT markers decreased, and epithelial markers E Cadherin expression increased. Cell proliferation, self-renewal ability, tumorigenesis and the ability to migrate were all restrained in AR-S/P cells. The characteristics of EMT were restrained too. These data suggest that AR played a dual inhibition effect on S/P cell proliferation and EMT features.

Signaling pathways regulating prostate cancer cell EMT included AKT, Wnt, stat3, Bcl-2, C-Myc, TGF-ß, EGF/EGFR, Notch, MAPK and so on [33-36]. It was shown that EMT and CSC characteristics could be regulated simultaneously. Erismodegib (a Shh signaling pathway inhibitor) could inhibit EMT and human prostate cancer stem cell growth in NOD/SCID IL2Rγ null mice by regulating Bmi-1 and microRNA-128[37]. In this study we tested a large number of cell signaling pathways. P-AKT, Bcl-2 and C-Myc expression were higher in S/P cells than those in non S/P cell. When AR was transferred into S/P cells, these three proteins expression were significantly suppressed. Then we designed SiBcl-2 and Si C-Myc and they could well restrain S/P cell proliferation. So it was suggested that P-AKT, Bcl-2 and C-Myc were involved in this procedure.

AR and the three signaling pathways can regulate S/P cells proliferation. So to intervene of these pathways, LY 294002, an inhibitor of AKT signaling, γ-TT, and 5-AZA, a de-methylating agent, were used. It was showed that γ-TT suppressed S/P cells through the interruption of targeting molecules including bcl-2 [38]. In addition, γ-TT has also been reported to inhibit PI3K/Akt signaling [39]. Therefore, it is possible that γ-TT might be able to suppress S/P cells via inhibiting both PI3K/Akt signaling and bcl-2 expression. The other strategy to suppress S/P cells is to target AR directly via reversing the low expression of AR in S/P cells. It was reported that the CpG islands in the promoter and exon 1 of AR gene in S/P cells were highly methylated, compared to that of the non S/P cells [40], and treatment of S/P cells with the de-methylating agent, 5-AZA, led to significant suppression of S/P cells proliferation via inducing AR expression. Combining two or three of the reagents could inhibit S/P cells tumorigenicity and growth efficiently. So, it was suggested that targeting AR or related cell signaling pathways could inhibit prostate cancer S/P cells proliferation, which could be a new strategy to treat castration resistant prostate cancer (CRPC).

## Conclusion

Our data showed that AR played a negative role in EMT of PCa S/P cells, by regulating AKT cell signaling pathway, which could be a new strategy to treat castration resistant prostate cancer (CRPC).

## Abbreviations

AR: Androgen receptor
FACS: Florescence-activated cell sorting
CRPC: Castration resistant prostate cancer
